# Rapid Review: Die Lebensqualität nach adulter allogener Nierentransplantation in den letzten 5 Jahren: Was können wir lernen?

**DOI:** 10.1007/s00120-024-02497-y

**Published:** 2025-01-07

**Authors:** Lujza Brunaiova, Stefanie Cermak, Lukas Koneval, Beat Roth, Laila Schneidewind

**Affiliations:** 1 Universitätsklinik für Urologie, Universität Bern, Inselspital Bern, Bern, Schweiz; 2 Universitätsklinik für Urologie, Universität Bern, Inselspital, Wilhelm-Fabry-Haus, Freiburgstr. 37, 3010 Bern, Schweiz

**Keywords:** Adulte Nierentransplantation, Lebensqualität, Ergebnisse, Transplantatüberleben, Nierenfunktion, Adult kidney transplantation, Quality of life, Outcomes, Graft survival, Kidney function

## Abstract

**Hintergrund:**

In aktuellen Studien konnte gezeigt werden, dass das klinische Monitoring der Lebensqualität (HRQoL) frühzeitig hilft, Nierentransplantatversagen zu erkennen.

**Fragestellung:**

Aufgrund des Potenzials, das in der Verbesserung der HRQoL für die Langzeitergebnisse der Nierentransplantation steckt, führten wir ein Rapid Review der letzten 5 Jahre zur Evaluation der Lebensqualität nach adulter allogener Nierentransplantation durch.

**Material und Methoden:**

Es wurde eine schnelle Evidenzanalyse mittels Literaturrecherche in MEDLINE im Zeitraum 2019 bis 2024 durchgeführt.

**Ergebnisse:**

Die primäre Literatursuche ergab 554 Treffer, schließlich konnten lediglich 12 Kohortenstudien eingeschlossen werden, davon 2 retrospektive und zehn prospektive Arbeiten. Nierentransplantierte Patienten haben eine bessere HRQoL als Patienten mit terminaler Niereninsuffizienz. Das HRQoL wird durch körperliche, psychische und soziale Faktoren beeinflusst. Eine weitere Verbesserung der HRQoL bzw. der primären Einflussfaktoren hat das Potenzial, die Ergebnisse der Nierentransplantation weiter zu verbessern. Allerdings fehlen Studien zur Identifikation geeigneter Interventionen. Interessante zu beeinflussende Faktoren könnten z. B. Atemwegsbeschwerden und Unterstützung bei der beruflichen Wiedereingliederung sein.

**Diskussion:**

Zukünftige Studien sollten auf die Identifikation adäquater Interventionen zur weiteren Verbesserung der HRQoL bei Nierentransplantierten fokussieren.

## Abstract (en)

**Background:**

Recent studies have also shown that clinical monitoring of quality of life (HRQoL) helps to recognize kidney transplant failure at an early stage.

**Objectives:**

Given the potential of improving HRQoL for the long-term outcomes of kidney transplantation, we conducted a rapid review of the last 5 years of quality of life evaluation after adult allogeneic kidney transplantation.

**Materials and methods:**

A rapid evidence analysis was carried out using a literature search in MEDLINE in the period 2019–2024.

**Results:**

The primary literature search yielded 554 hits, and ultimately only 12 cohort studies could be included, of which 2 were retrospective and 10 prospective cohort studies. Kidney transplant patients have better HRQoL than patients with end-stage renal disease. HRQoL is influenced by physical, psychological, and social factors. Further improvement of HRQoL or the primary influencing factors has the potential to further enhance the outcomes of kidney transplantation. However, studies to identify suitable interventions are lacking. Interesting factors to be influenced could be, for example, respiratory symptoms and support for professional reintegration.

**Conclusion:**

Future studies should focus on the identification of adequate interventions to further improve HRQoL in kidney transplant recipients.

## Hintergrund und Fragestellung

Bei der Nierentransplantation handelt es sich um die bevorzugte Therapie der terminalen Niereninsuffizienz, insbesondere da sie das Überleben sowie die Lebensqualität der Patienten im Vergleich zu anderen Nierenersatzverfahren erheblich verbessert [1]. In neueren Untersuchungen konnte weiterhin gezeigt werden, dass das klinische Monitoring der Lebensqualität frühzeitig hilft, Transplantatversagen zu erkennen und damit die Patienten früher einer entsprechenden Therapie zuzuführen sowie, dass insbesondere ältere Patienten hinsichtlich der Lebensqualität von der Nierentransplantation profitieren [2, 3]. Vor dem Hintergrund der aktuellen demographischen Entwicklung ist dieses Ergebnis von übergeordneter Bedeutung.

Das kontrovers diskutierte Organspendegesetz und die langen Wartezeiten auf eine Nierentransplantation in Deutschland machen es noch essentieller, die Ergebnisse zu verbessern, um das Vertrauen in der Bevölkerung für dieses Verfahren zu stärken. Weiterhin sind die Lebensqualität sowie die umfassende ganzheitliche, interdisziplinäre medizinische Betreuung nach der Nierentransplantation entscheidend für das Langzeitüberleben des Transplantats [4]. Leider ist die Analyse und Verbesserung der Lebensqualität durch frühe Intervention zumindest in der deutschen Nierentransplantationsforschung und -klinik noch unterrepräsentiert [5].

Aufgrund dieser Datenlage sowie dem Potenzial, das in der Verbesserung der Lebensqualität für die Langzeitergebnisse der Nierentransplantation steckt, führten wir ein Rapid Review der letzten 5 Jahre zur Evaluation der Lebensqualität nach adulter allogener Nierentransplantation durch. Ein besonderer Fokus sollte dabei auf Interventionen liegen, die die Lebensqualität in der klinischen Praxis verbessern können.

## Methodik

Es wurde eine schnelle Evidenzanalyse mit Literaturrecherche in MEDLINE via PubMed für den Zeitraum Januar 2019 bis zum Datum der letzten Suche (15. Juli 2024) durchgeführt [6]. Als Suchbegriffe wurden die Begriffe „quality of life“ und „kidney transplantation“ verwendet. Für die Evidenzsynthese wurden lediglich Originalarbeiten genutzt, Fallberichte sowie Übersichtsarbeiten wurden nicht berücksichtig. Weiterhin wurden nur Untersuchungen mit adulten Patienten eingeschlossen. Bezüglich der Sprache wurden nur englische und deutsche Arbeiten verwendet. Der primäre Endpunkt dieser Arbeit ist die Deskription der Lebensqualität nach adulter allogener Nierentransplantation. Sekundäre Endpunkte stellen Interventionen zur Verbesserung der Lebensqualität nach diesen Verfahren sowie Implikationen für die klinische Praxis und Forschung dar. Weiterhin wurden die PRISMA-Leitlinien zur Berichterstattung systematischer Übersichtsarbeiten angewandt [7]. Die Qualitätsbewertung der inkludierten Studien erfolgte mit dem ROBINS-I-Instrument [8].

## Ergebnisse

**Abb. 1:**
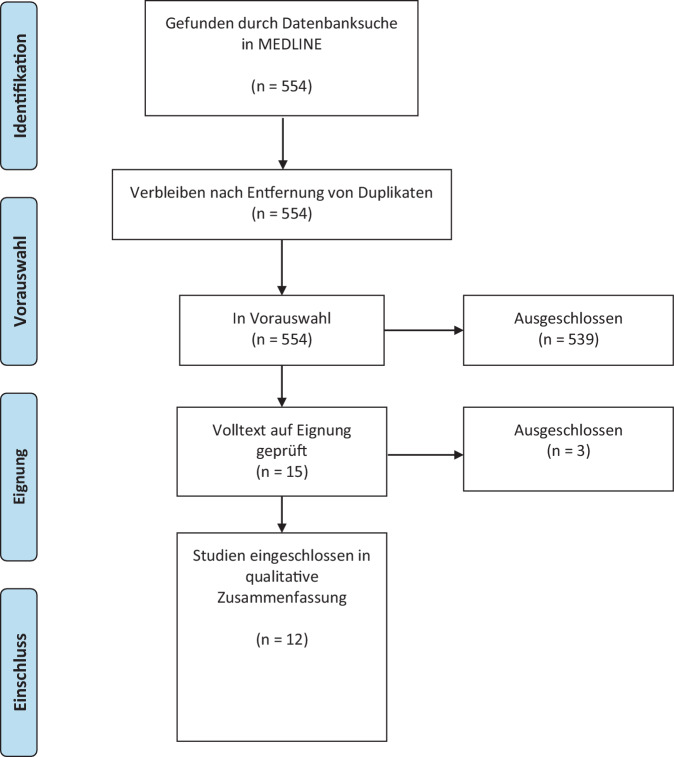
PRISMA-Flussdiagramm

Die primäre Literatursuche ergab 554 Treffer, schließlich konnten lediglich 12 Kohortenstudien eingeschlossen werden, davon 2 retrospektive und 10 prospektive Arbeiten (Abb. 1: PRISMA-Flussdiagramm). Die Tab. 1 gibt einen Überblick über die eingeschlossenen Studien sowie deren Charakteristika.

**Tab. 1:** Überblick und Charakterisierung der eingeschlossenen Studien (*n* = 12)

Referenz	Studiendesign	Untersuchte Population	Benutzte Fragebögen/Tests	Primärer Endpunkt	Sekundäre Endpunkte	Hauptergebnisse	Schlussfolgerung der Autoren
De Boer S et al. 2024 [3]	Retrospektive Kohortenstudie, unizentrisch, Daten aus der Transplantlines-Biobank and Kohortenstudie in den Niederlanden, Zeitraum: 2015 – unklar	145 Patienten auf der Transplantatwarteliste (68 % Männer, Alter 70 ± 4 Jahre) und 115 nierentransplantierte Patienten (73 % Männer, Alter 70 ± 4 Jahre)	SF-36	Vergleich der HRQoL von Patienten auf der Tx-Liste und Patienten nach der Tx	1. HRQoL mit standardisiertem MCS und PCS 1 Jahr nach Nieren-Tx 2. HRQoL von Teilnehmern auf der Tx-Liste 3. Identifikation von Fakten, welche die HRQoL nach der Tx beeinflussen	Das MCS (48,5 ± 8,4 vs. 51,2 ± 7,7, *p* = 0,009) sowie PCS (47,4 ± 8,5 vs. 52,1 ± 7,2, *p* > 0,001) der HRQoL war besser bei Tx-Patienten, verglichen mit den Patienten auf der Tx-Liste. In der Paaranalyse von 46 Patienten mit dokumentierter HRQoL vor und nach der Tx hat sich eine Besserung in beiden Komponentenskoren gezeigt (MCS 49,1 ± 8,4 zu 51,6 ± 7,5, *p* = 0,054; PCS 48,1 ± 8,0 zu 52,4 ± 6,7, *p* = 0,001). Von allen untersuchten Faktoren waren die Immunsuppressiva-assoziierte Nebenwirkungen 1 Jahr nach Tx am stärksten mit der MCS und PCS HRQoL negativ assoziiert	Die HRQoL ist besser bei nierentransplantierten Patienten im Vergleich zu Patienten auf der Tx-Liste. Nach Nieren-Tx haben die Patienten bessere HRQoL als vor der Nieren-Tx
Pozza G et al. 2020 [9]	Retrospektive, Kohortenstudie, unizentrisch (Padua, Italy) Zeitraum: 01/2005–12/2015 mit Follow-up von 47 Monaten (zwischen 18 und 188)	19 Nierentransplantierte mit Blasenaugmentation oder Harnableitung	SF-36	HRQoL resp. einzelne Subdomänen Häufigkeit der akuten Harnwegsinfektion Häufigkeit der asymptomatischen Bakteriurie	Gesamtüberleben Kreatinin	Die Tx-Patienten erzielten niedrigere Werte als die italienische Allgemeinbevölkerung für PF (*p* < 0,0001), PR (*p* = 0,0007), BP (*p* = 0,0046), GH (*p* < 0,0001), RE (*p* = 0,0462) und SF (*p* = 0,0200). Es gab keinen statistisch signifikanten Unterschied für VT (*p* = 0,8088) und MH (*p* = 0,8668). Gesamtüberleben nach der NTx 94,8 %, Transplantatüberleben 89,6 %, mittlerer Kreatininwert 1 Jahr nach der Tx 102 μmol/l, rezidivierende HWI in 23,5 % vs. 18,8 % bei Nieren-Tx ohne Rekonstruktion von unteren Harntrakt, asymptomatische Bakteriurie (53 %)	Die nierentransplantierten Patienten mit Blasenaugmentation oder einer anderen Funktionsstörung der unteren Harnwege haben kein schlechteres Endergebnis bei der Nierentransplantation. Die psychische Gesundheit und Vitalität dieser Patienten ähneln denen der Allgemeinbevölkerung
Ziengs A et al. 2023 [10]	Prospektiv Kohortenstudie, unizentrisch, Daten aus der Transplantlines-Biobank and Kohortenstudie in den Niederlanden, Zeitraum: 2015 – unklar	131 nierentransplantierte Patienten und 306 gesunde Teilnehmer in der Kontrollgruppe	Digital Span Forward 15 Words Test Word Fluency Trail Making Test part A Trail Making Test part B Controlled Oral Word Association Test Digit Span Backward USER‑P SF-36	Vergleich von langfristiger (11 Jahre nach Tx) neurokognitiven Funktion bei Tx-Patienten und bei gesunder Kontrollgruppe	Identifikation von Faktoren, die mit neurokognitivem Defizit verbunden sind: krankheitsassoziierte Variablen. Assoziation zwischen neurokognitivem Defizit und geringerer gesellschaftlichen Teilnahme sowie geringerer HRQoL	Bei 16 % von Tx-Patienten wurde ein MCI diagnostiziert. In der Kontrollgruppe war es nur 2,6 % (*p* < 0,001). Keine Korrelation zwischen MCI und krankheitsassoziierten Faktoren. Die gesellschaftliche Teilnahme, die durch USER‑P untersucht wurde, hat sich signifikant kleiner in der Tx-Gruppe gezeigt. Die Lebensqualität war bei Tx-Patienten signifikant schlechter als bei HC (für PCS *p* < 0,001, für MCS *p* < 0,001)	Die Tx-Patienten haben eine langfristige, kognitive Beeinträchtigung. Die frühzeitige neuro-psychologische Beurteilung ist wichtig, da die kognitive Beeinträchtigung einen negativen Effekt an die gesellschaftliche Teilnahme und HRQoL haben kann
Wang Y et al. 2023 [11]	Prospektive Kohortenstudie, multizentrisch (2 Zentren in den Niederlande), Teil von der „POSITIVE»“ Studie (The Patient-reported Outcomes In kidney Transplant recipients: Input of Valuable Endpoints), Zeitraum: 2019 – unklar (Originalstudie rekrutiert weiter)	90 nierentransplantierte Patienten und allgemeine holländische Population als Kontrollgruppe	SF-12v2 mit PCS und MCS Dialysis Symptom Index (DSI) Modified Transplant Symptom Occurrence and Symptom Distress Scale-59 Items Revised (MTSOSD-59r) Brief Illness Perception Questionnaire (Brief-IPQ)	HRQoL während Transplantation und 6 Wochen nach der Transplantation	Ist die HRQoL während Transplantation und 6 Wochen nach der Transplantation durch die Krankheitswahrnehmungen und durch das Erleben von Symptomen (Auftreten von Symptomen und Belastungsgrad des Patienten durch die Symptome) beeinflusst?	6 Wochen nach der Transplantation: Ein signifikanter Unterschied in MCS im Vergleich zur vor der Tx (49,9 ± 10,7 vs. 44,7 ± 10,7) aber kein signifikanter Unterschied im Vergleich zur Kontrollgruppe (50,2 ± 9,2, *p* = 0,76); bezüglich PCS: kein Unterschied zwischen peritransplantationell und 6 Wochen nach der Tx aber signifikant schlechter als bei der Kontrollgruppe (38,9 ± 9,1 vs. 39,9 ± 9,6 vs. 50,6 ± 9,2). Mit jedem einzelnen Symptom während der Tx nimmt die mentale HRQoL nach der Tx statistisch signifikant ab (−0,23 pro Symptom). Der steigende Belastungsgrad beeinflusst die mentale und physische HRQoL nicht signifikant	Das Erleben von Symptomen während Transplantation kann die HRQoL 6 Wochen nach der Tx beeinflussen, wobei dieser Einfluss teilweise durch die Krankheitswahrnehmungen von jedem einzelnen Patienten vermittelt wird. Die Autoren schlagen ein aktives Management von Symptomen vor sowie eine Modifikation von nicht hilfreichen Krankheitswahrnehmungen
Sawada A et al. 2021 [12]	Prospektive Kohortenstudie, unizentrisch in Tokyo, Zeit zwischen Nieren-Tx und Einschluss in die Studie: mittlere Zeit 106 Monate (1–426Monate)	67 nierentransplantierte Patienten	Kidney Disease Quality of Life-Short Form (KDQoL-SF), version 1.3 (SF-36 inbegriffen) EuroQoL‑5 dimension-5 levels (EQ-5D-5L)	Effekt von jeder KDQoL-SF Domäne auf das EQ-5D-5L bei Patienten nach Nieren-Tx	–	Die körperliche Funktionsfähigkeit (r = 0,749) und körperliche Alltagsfunktionsfähigkeit (r = 0,603) zeigten eine starke Korrelation mit EQ-5D-5L. Die Domänen, die sich auf die psychologischen und sozialen Aspekte der Lebensqualität beziehen, zeigten eine nicht signifikante Korrelation. Die nierenkrankheitsspezifische Symptome/Probleme zeigten im Zusammenhang mit der körperlichen Funktion eine gute Korrelation (r = 0,691) mit EQ-5D-5L. Die anderen Skalen, einschließlich der Belastung durch die Nierenerkrankungen (r = 0,168), der Qualität der sozialen Interaktion (r = 0,284) und den mentalen und sozialen Aspekten der Lebensqualität, zeigten eine geringe Korrelation mit EQ-5D-5L	Die körperlichen Gesundheitsaspekte der Lebensqualität (Symptome/Probleme) waren die Hauptfaktoren, die die allgemeine Lebensqualität beeinflussten
Sarhan A et al. 2021 [13]	Prospektive Kohortenstudie, multizentrisch (2 Zentren in Palästina); Zeitraum: 05–08/2017; bei ausfüllen des Fragebögen waren die dialysierten Patienten mind. 3 Monate unter Dialyse und die Tx-Patienten mind. 1 Jahr nach Tx	100 nierentransplantierte Patienten und 272 hämodialysierte Patienten	SF-36	Unterschiede in der mittleren HRQoL zwischen den nierentransplantierten und hämodialysierten Patienten	Soziodemographische Unterschiede und Unterschiede im Geschlecht und Alter beider Gruppen	Die Tx-Patienten haben eine signifikant bessere HRQoL im Vergleich zu Patienten an der Hämodialyse und das in allen Domänen der SF-36 (Der Unterschied in PCS ist 28,6 und in MCS 22,5)	Die Tx-Patienten haben eine bessere HRQoL als die hämodialysierten Patienten in den physischen sowie mentalen Domänen der SF-36
Knobbe T et al. 2021 [14]	Prospektive Kohortenstudie, unizentrisch, Daten aus der Transplantlines-Biobank and Kohortenstudie in den Niederlanden, Zeitraum: 2015–2020	539 nierentransplantierte Patienten und 244 gesunde Kontrollgruppe (gesunde Nierenspender)	Asma‑1 Spirometer (Vitalograph, Buckingham, UK) CIS20‑R (checklist individual strength 20 revised) SF-36	Prävalenz der Atembeschwerden unter nierentransplantierten Patienten und Einfluss der Atembeschwerden auf die Müdigkeit und HRQoL in dieser Population	Prävalenz von Komorbiditäten bei Tx-Patienten mit Atembeschwerden	Die Prävalenz der Atembeschwerden war bei Tx-Patienten höher als in der Kontrollgruppe (25 % vs. 10 %) Atemwegsbeschwerden waren mit dem Schwergrad der Müdigkeit und Verschlechterung der PCS assoziiert. In multinomialen Regressionsmodellen hat sich eine Assoziation zwischen Atembeschwerden und moderater Müdigkeit (OR 1,68 mit 95 %-KI 0,92–3,09) und starke Müdigkeit (OR 2,51 mit 95 %-KI 1,39–4,55, *p* = 0,007) sowie schlechterer physischer HRQoL (−0,11 SDs, 95%-KI −0,19 bis −0,02, *p* = 0,01) gezeigt. Diabetes mellitus, COPD, OSAS, erhöhte NB-proBNP und Dialyse vor der Transplantation waren assoziiert mit Atembeschwerden bei nierentransplantierten Patienten	Atemwegsbeschwerden sind bei Tx-Patienten häufig und ihr Auftreten erhöht 2 × das Risiko für schwerer Müdigkeit und ist mit Verschlechterung von physischer HRQoL assoziiert
El-Agroudy, AE et al. 2021 [15]	Prospektive, Kohortenstudie, unizentrisch (Bahrain), Zeitraum: 06/2019–11/2019	58 nierentransplantierte Patienten	QOLI	QoL nach Tx	Unterschied in der familiären Domäne bei verheirateten und ledigen Patienten Einfluss der QoL durch Diabetes mellitus	Die höchsten QOL-Werte wurden im psychologischen/spirituellen Bereich (87,4 ± 12,2) erzielt, gefolgt vom familiären Bereich (85,5 ± 13,1), dem Bereich Gesundheit/Funktionsfähigkeit (82,7 ± 13,3) und dem sozialen/wirtschaftlichen Bereich (80,5 ± 13,9). Positive Korrelation zwischen der QOLI und jedem der getesteten Bereiche (*p* < 0,001)	Die QOLI sowie ihre vier Domänen zeigten keinen signifikanten Zusammenhang mit dem Alter, Geschlecht, Bildungsniveau, monatliches Einkommen, Beschäftigungsstatus, Jahr der Transplantation, Quelle der transplantierten Niere und Gesamtdauer der Dialyse vor der Nierentransplantation
Rocha FL et al. 2020 [16]	Prospektive Kohortenstudie, unizentrisch, Zeitraum: 10/2016–02/2017	47 nierentransplantiertePatienten	SF-36 Beck Depression Inventory Rosenberg Self-Esteem Scale	Zusammenhang zwischen der HRQoL und Depressionen sowie Selbstwertschätzung nach Nieren-Tx	Unterschieden zwischen Männer und Frauen in allen Domänen des SF-36-Fragebogens	Frauen erzielten schlechtere HRQoL. Junge Erwachsene, nierentransplantierte Patienten mehr als 1,5 Jahre postoperativ und Patienten, die wurden präoperativ hämodialysiert hatten bessere HRQoL	Die HRQoL war gut bis ausgezeichnet. Depressionen wurden nicht nachgewiesen. Je höhere HRQoL desto bessere Selbstwertschätzung
Ranabhat K et al. 2020 [17]	Prospektive Kohortenstudie, multizentrisch (2 Zentren in Nepal), Zeitraum: 10–11/2018	161 Teilnehmen (92 nierentransplantierte Patienten und 69 dialysierte Patienten)	WHOQoL-BREF	Vergleich der HRQoL von Patienten transplantierten und dialysierten Patienten	Faktoren, die die HRQoL in jeder Gruppe beeinflussen	Die dialysierten Patienten hatten schlechtere allgemeinte HRQoL (*p* < 0,001) sowie schlechtere Ergebnisse in allen 4 Gesundheitsdomänen des Fragebogens im Vergleich zu NTx-Patienten (physische Gesundheit *p* < 0,001, psychologische Gesundheit *p* < 0,001, soziale Beziehungen 0,012, Umwelt 0,004). Ethnische Zugehörigkeit (*p* = 0,020), sozioökonomische Status (*p* < 0,001), Bildungsstatus (*p* < 0,001) und Beschäftigungsstatus (*p* = 0,009) waren signifikant mit der allgemeinen HRQoL bei ESRD-Patienten verbunden. Bei Dialysepatienten beeinflusst das Bildungsstatus die HRQoL positiv (*p* = 0,012). Bei Tx-Patienten war ein Wohnsitz in der Stadt (*p* = 0,023), ein höherer sozio-ökonomischer Status (*p* < 0,001), ein höherer Bildungsstatus (*p* = 0,004) und Diabetesstatus (*p* = 0,010) signifikant mit einer besseren HRQoL verbunden	Die allgemeine HRQoL sowie die HRQoL in allen vier Bereichen des WHOQOL-BREF von NTx-Patienten war höher als den Patienten unter Hämodialyse. Die Patienten mit terminaler Niereninsuffizienz und niedriger HRQoL könnten von gezielten risikomodifizierenden Interventionen profitieren
Peipert JD et al. 2020 [2]	Prospektive Kohortenstudie, unizentrisch, Kohortenstudie, Zeitraum: 11/2007–08/2016	477 NTx-Patienten mit Lebendspende	SF-36 Kidney Disease Quality of Life-Short Form (KDQOL-SF) Functional Assessment of Cancer Therapy—Kidney Symptom Index 19 item version (FKSI-19)	Änderung von HRQoL (vor der NTx, 3 und 12 Monate nach der NTx)	Zusammenhang zwischen HRQOL-Trends und Überleben des Transplantats	Die NTx hatte eine große Auswirkung (d > 0,80) auf die postoperative Besserung der Vitalitätsskala vom SF-36 (d = 0,81) sowie der Subdomäne Belastung durch Nierenerkrankungen des KDQOL-SF (d = 1,05). Kleine Kreatininbesserung sowie höher Alter sind mit kleinerer Besserung von HRQoL verbunden	HRQoL – Überwachung kann das Risiko von Transplantatversagen und von Notwendigkeit einer Post-NTx Intervention einschätzen
JordakievaG et al. 2020 [18]	Prospektive Kohortenstudie, multizentrisch (Wien und Graz), Zeitraum: 2012 – unklar	139 NTx-Patienten	Brief Symptom Inventory (BSI-18) World Health Organization Quality of Life (WHOQOL-Brief) Workability Index (WAI)	Zusammenhang zwischen Lebensqualität oder der psychischen Gesundheit von NTx-Patienten, und dem Arbeitsverhältnis nach NTx	Einfluss von Bildungsgrad, Partnerschaft, Alkoholkonsum auf das Berufsleben nach NTx	Berufstätige Patienten lebten häufiger in einer Partnerschaft (*p* = 0,018), hatten einen höheren Bildungsgrad (*p* = 0,01) und bessere HRQoL (*p* < 0,001). Arbeitslose NTx-Patienten hatten mehr Müdigkeit und psychische Probleme (*p* < 0,001) und wiesen signifikant höhere Angst‑, Depressions- und Somatisierung auf (BSI-18)	Die berufliche Rehabilitations- und RTW-Programme sollten sich auf eine verstärkte soziale Unterstützung und die Sorge um die psychische Gesundheit konzentrieren, um den Wiedereinstieg ins Berufsleben zu erleichtern

*HRQoL* „health related quality of life“, gesundheitsbezogene Lebensqualität, *MCS* Mental Component Score, *PCS* Physical Component Score, *NTx* Nierentransplantation, *NTx-Liste* Nierentransplantationswarteliste, *MCI* „minimal cognitive impairment“, *HC* „healthy control“, gesunde Kontrollgruppe, *r* Korrelationskoeffizient nach Pearson, *ESRD* „end stage renal disease“, terminale Niereninsuffizienz, *RTW* „return to work“

### Ergebnisse der retrospektiven Kohortenstudie

Es konnten zwei retrospektive Arbeiten für diese Übersichtsarbeit mit insgesamt 164 Patienten verwendet werden. Beide Studien verwendeten den SF-36 als validiertes Messinstrument der „health-related quality of life“ (HRQoL). Es konnte gezeigt werden, dass Nierentransplantierte im Vergleich zu Patienten auf der Transplantationswarteliste und im Vergleich zum Zeitpunkt vor der Transplantation eine bessere HRQoL haben [3]. Weiterhin haben nierentransplantierte Patienten mit Blasenaugmentation oder anderen Funktionsstörungen der unteren Harnwege kein schlechteres Endergebnis bzgl. der HRQoL als andere Nierentransplantierte [9].

### Ergebnisse der prospektiven Kohortenstudien

Insgesamt konnten 10 prospektive Kohortenstudien mit 1740 Nierentransplantierten für dieses Rapid Review identifiziert werden [2, 10–18].

Interessanterweise verwenden 6 der inkludierten Untersuchungen den SF-36 zur Beurteilung der HRQoL [2, 10, 12–14, 16]. Essentielle Ergebnisse zeigen hier, dass nierentransplantierte Patienten eine langfristige kognitive Beeinträchtigung im Vergleich zu einer gesunden Kontrollgruppe haben, die einen negativen Effekt auf die Teilnahme am gesellschaftlichen Leben als auch auf die HRQoL hat [10]. Außerdem haben die körperlichen Aspekte der Lebensqualität den größten Einfluss auf die HRQoL bei diesen Patienten [12, 14] und im prospektiven Setting konnte wiederum gezeigt werden, dass Nierentransplantierte im Vergleich zu Patienten an der Hämodialyse eine signifikant bessere HRQoL aufweisen [13].

### Interventionen und Implikationen für die klinische sowie wissenschaftliche Praxis

Es konnte keine Interventionsstudie zur HRQoL bei Nierentransplantierten identifiziert werden. Allerdings schlussfolgerten 5 der eingeschlossenen prospektiven Studien, dass Nierentransplantierte von psychologischer Beurteilung sowie Intervention profitieren können und damit psychologisch betreut werden sollten [2, 10, 11, 17, 18].

Die stringente Überwachung von HRQoL hat das Potenzial, das Risiko von Transplantatversagen abzuschätzen [2]. Zusätzlich konnten Jordakieva et al. zeigen, dass arbeitslose nierentransplantierte Patienten signifikant häufiger unter psychischen Problemen litten als Berufstätige, insbesondere hinsichtlich Angst und Depression. Somit schlussfolgerten die Autoren, dass sich berufliche Rehabilitationsprogramme verstärkt auf soziale Unterstützung sowie die Sorge um die psychische Gesundheit konzentrieren sollten, um den Transplantierten einen Wiedereinstieg in das Berufsleben zu ermöglichen und damit ebenfalls deren HRQoL weiter zu verbessern [18].

### Qualitätsbewertung der inkludierten Studien

Die Tab. 2 gibt einen Überblick über die Qualitätsbewertung der eingeschlossenen Studien. Insgesamt hat 1 Studie ein hohes Risiko, 5 Studien ein moderates Risiko und 6 Studien ein niedriges Gesamtrisiko für Bias, so dass von einer zufriedenstellenden Studienqualität gesprochen werden kann.

**Tab. 2:** Qualitätsbewertung der inkludierten Studien mit dem ROBINS-I-Instrument (*n* = 12)

Referenz	Risk of bias due to confounding	Bias in selection of participants into the study	Bias in classification of interventions	Bias due to deviations from intended interventions	Bias due to missing data	Bias in measurement of outcomes	Bias in selection of the reported results	Gesamtrisiko für Bias
De Boer S et al. 2024 [3]	Moderates Risiko	Moderates Risiko	Niedriges Risiko	Niedriges Risiko	Niedriges Risiko	Niedriges Risiko	Niedriges Risiko	Moderates Risiko
Pozza G et al. 2020 [9]	Moderates Risiko	Moderates Risiko	Moderates Risiko	Niedriges Risiko	Niedriges Risiko	Niedriges Risiko	Moderates Risiko	Moderates Risiko
Ziengs A et al. 2023 [10]	Niedriges Risiko	Niedriges Risiko	Niedriges Risiko	Niedriges Risiko	Niedriges Risiko	Niedriges Risiko	Niedriges Risiko	Niedriges Risiko
Wang Y et al. 2023 [11]	Moderates Risiko	Moderates Risiko	Niedriges Risiko	Niedriges Risiko	Niedriges Risiko	Moderates Risiko	Moderates Risiko	Moderates Risiko
Sawada A et al. 2021 [12]	Niedriges Risiko	Niedriges Risiko	Moderates Risiko	Niedriges Risiko	Moderates Risiko	Niedriges Risiko	Niedriges Risiko	Moderates Risiko
Sarhan A et al. 2021 [13]	Niedriges Risiko	Niedriges Risiko	Moderates Risiko	Niedriges Risiko	Niedriges Risiko	Niedriges Risiko	Moderates Risiko	Moderates Risiko
Knobbe T et al. 2021 [14]	Niedriges Risiko	Niedriges Risiko	Niedriges Risiko	Niedriges Risiko	Niedriges Risiko	Niedriges Risiko	Niedriges Risiko	Niedriges Risiko
El-Agroudy, AE et al. 2021 [15]	Moderates Risiko	Moderates Risiko	Hohes Risiko	Hohes Risiko	Hohes Risiko	Hohes Risiko	Hohes Risiko	Hohes Risiko
Rocha FL et al. 2020 [16]	Niedriges Risiko	Niedriges Risiko	Niedriges Risiko	Niedriges Risiko	Niedriges Risiko	Niedriges Risiko	Niedriges Risiko	Niedriges Risiko
Ranabhat K et al. 2020 [17]	Niedriges Risiko	Niedriges Risiko	Niedriges Risiko	Niedriges Risiko	Niedriges Risiko	Niedriges Risiko	. Niedriges Risiko	Niedriges Risiko
Peipert JD et al. 2020 [2]	Niedriges Risiko	Niedriges Risiko	Niedriges Risiko	Niedriges Risiko	Niedriges Risiko	Niedriges Risiko	Niedriges Risiko	Niedriges Risiko
Jordakieva, G et al. 2020 [18]	Niedriges Risiko	Niedriges Risiko	Niedriges Risiko	Niedriges Risiko	Niedriges Risiko	Niedriges Risiko	Niedriges Risiko	Niedriges Risiko

## Diskussion

In der durch uns durchgeführten schnellen Evidenzanalyse konnte anhand von 12 eingeschlossenen Studien gezeigt werden, dass nierentransplantierte Patienten eine bessere HRQoL als Patienten vor der Nierentransplantation, insbesondere unter Hämodialyse, haben. Außerdem wurde dargestellt, dass die HRQoL und die allgemeine Lebensqualität durch körperliche, psychische und soziale Faktoren beeinflusst wird. Leider konnten keine Interventionsstudien hinsichtlich der HRQoL nach Nierentransplantation identifiziert werden, welche die einzelnen, lebensqualitätbeeinflussenden Faktoren untersuchen. Hinsichtlich des Studiendesigns konnten 12 Kohortenstudien identifiziert werden.

In der retrospektiven Studie von De Boer et al. aus dem Jahr 2024 mit 115 Nierentransplantierten konnte gezeigt werden, dass die Immunsuppressiva-assoziierten Nebenwirkungen von allen untersuchten Faktoren ein Jahr nach der Nierentransplantation am stärksten die HRQoL negativ beeinflussen [3]. Zu den untersuchten Faktoren haben u. a. Alter, Geschlecht, präventive Nierentransplantation, Transplantatabstoßung und erneute Hospitalisation innerhalb von einem Jahr nach der Transplantation gehört. Von daher kann postuliert werden, dass eine Optimierung und bessere Einstellung der Immunosuppressivaeinnahme zur besseren HRQoL führen könnte. Zusätzlich wurde gezeigt, dass ein höheres Einkommen mit einem schlechteren physischen Zustand assoziiert war, was widersprüchlich zur prospektiven Kohortenstudie von Ranabhat et al. aus Nepal ist [17], in welcher eine Assoziation zwischen einem Wohnsitz in der Stadt (*p* = 0,023), einem höheren sozioökonomischen Status (*p* < 0,001) und einem höheren Bildungsstatus (*p* = 0,004) mit einer besseren HRQoL zeigten. Widersprüchliche Ergebnisse erbrachte auch eine prospektive, unizentrische Kohortenstudie aus Bahrain, die keinen signifikanten Zusammenhang mit dem Alter, Geschlecht, Bildungsniveau, monatliches Einkommen und Beschäftigungsstatus zeigte [15], so dass hier keine abschließende Aussage getätigt werden kann. Denkbar wäre ein Zusammenhang mit den lokalen sowie nationalen Gegebenheiten.

Außerdem ist bekannt, dass Nierentransplantierte im Vergleich zur normalen Population eine langfristige kognitive Beeinträchtigung haben. Die Kohortenstudie von Ziengs et al. dagegen, im Jahr 2023 publiziert, hat keine Assoziation zwischen multiplen krankheitsbezogenen Variablen (Nierenfunktion, Zeit an der Hämodialyse vor der Nierentransplantation, Art der Immunsuppression, Gesundheitszustand vor der Nierentransplantation – Zustand nach Herzinfarkt, Zustand nach Hirninfarkt oder Diabetes mellitus) und dem neuro-kognitiven Status identifiziert [10]. Eine Assoziation wurde aber bei neurokognitivem Status und geringerer gesellschaftlichen Teilnahme nachgewiesen, was schlussendlich zu einer geringeren HRQoL führte. Ähnliche Ergebnisse konnten in der Studie von Jordakieva et al. gezeigt werden, wobei arbeitstätige Patienten bessere HRQoL hatten. Frühzeitiges Screening von kognitiver Einschränkung, frühe soziale Unterstützung, berufliche Rehabilitation und Wiedereingliederungsprogramme könnten der Weg zur Besserung der HRQoL nach der Nierentransplantation sein [18].

Interessanterweise beschreibt die prospektive Kohortenstudie von Wang et al., dass mit jedem einzelnen zusätzlichen Symptom nach der Nierentransplantation, die mentale HRQoL statistisch signifikant abnimmt (−0,23 pro Symptom), wobei meisten erwähnten Symptome Müdigkeit, Energiemangel, Schwierigkeiten beim Einschlafen und Nykturie waren [11]. Eine weitere prospektive Kohortenstudie unterstrich ebenso die Wichtigkeit der körperlichen Gesundheitsaspekte der Lebensqualität [12]. Eine frühzeitige und patientenorientierte Symptomkontrolle bzw. deren Prävention sowie aktiver Patienteneinsatz bei Lösung von Komplikationen/Symptomen nach der Transplantation könnte zur Besserung der HRQoL führen.

Ein weiterer modifizierbarer Faktor ist bei den Nierentransplantierten die pulmonale Situation. Die eingeschlossene prospektive Kohortenstudie mit 539 Nierentransplantierten aus den Niederlanden hat eine größere Prävalenz von Atembeschwerden bei Nierentransplantierten im Vergleich zur normalen Population gezeigt [14]. Die beschriebenen Atembeschwerden waren mit einer Risikoverdoppelung für schwere Müdigkeit und Verschlechterung der physischen HRQoL assoziiert. Ein Screening der Atemerkrankungen und eine Optimierung der Atemsituation mit Therapie eines OSAS, einer COPD oder anderweitiger Atemwegsbeschwerden könnte zur Besserung des physischen Zustandes und auch der Lebensqualität führen.

Die allgemeinen psychosozialen Risikofaktoren bei soliden Organtransplantationen sind bekannt und in der entsprechenden AWMF-S3-Leitlinie erwähnt. Zusammenfassend handelt es sich v. a. um Adipositas, depressive Störungen und Angststörungen [19].

In den letzten Jahren wurden nur wenige Interventionsstudien bei Nierentransplantierten publiziert. Es ist bekannt, dass 13–40 % Patienten nach einer Organtransplantation durch eine Depression betroffen sind, wobei die Mortalitätsraten bei diesen Patienten um 65 % steigt [20]. Zu diesem Thema ist eine kleine, randomisiert kontrollierte Kohortenstudie aus Italien aus dem Jahr 2022 zu erwähnen, welche die Effizienz und den Einfluss von expressivem Schreiben auf die psychologischen und physiologischen Variablen bei Nierentransplantierten untersuchte. In der Interventionsgruppe gab es weniger Depressionen, weniger Alexithymie (Gefühlsblindheit), bessere Adhärenz zu pharmakologischer Therapie und so wahrscheinlich auch bessere Nierenfunktion [21]. Eine kleine, multizentrische Studie aus den USA mit 48 Teilnehmern, die 2020 publiziert wurde, hat eine signifikante Reduktion von Angstzuständen bei Patienten identifizieren können, die nach der Organtransplantation sog. „symptom-targeted interventions“ mittels speziellen Trainings mit kognitiv – behavioraler Therapie, Motivationsinterviews und Achtsamkeitstherapie erhalten haben [22].

Ein weiterer wichtiger Aspekt ist eine ausgewogene Ernährung nach der Nierentransplantation. Eine prospektive, randomisiert kontrollierte Studie aus Brasilien mit 120 Teilnehmern hat den Effekt von proteinreicher und zuckerarmer Ernährung nach der Nierentransplantation untersucht. Die Studie konnte keinen Einfluss der Nahrungsumstellung auf die Gewichtszunahme im Vergleich zu normal ernährten Patienten aufzeigen [23]. Eine weitere kleine Kohortenstudie aus Japan hat eine Proteinaufnahme von mindestens 0,72 g/kg ideales Körpergewicht/Tag empfohlen, v. a. bei Nierentransplantierten, die unter einem Verlust an Skelettmuskelmasse leiden [24]. Internationale Empfehlungen schlagen ebenfalls eine Proteinaufnahme von 0,8 g/kg Körpergewicht pro Tag bei Erwachsenen vor [25]. Hingegen haben in einer randomisierten kontrollierten Studie aus Neuseeland die Nierentransplantierten im ersten Jahr nach der Transplantation nicht von einer intensiven Ernährungsintervention im Vergleich zur Standardernährungsversorgung profitiert [26]. In einer älteren Studie aus dem Jahr 2007 hat sich ein Zusammenhang zwischen diätetischen Maßnahmen, reduziertem Körperfett, Gewichtsverlust, niedrigerem Cholesterin- und Triglycerinspiegel sowie einer Verbesserung des Glukosespiegels gezeigt [27]. Inwiefern die Ernährung allerdings konkret die HRQoL von Nierentransplantierten beeinflusst, muss zunächst unbeantwortet bleiben, sollte aber unbedingt Gegenstand zukünftiger Untersuchungen sein.

Die Adhärenz zur medikamentösen, immunsuppressiven Therapie nach solider Organtransplantation bleibt ein persistierendes Problem, welches ca. 25–30 % der Organtransplantierten betrifft. Malcompliance scheint mit einem erhöhten Risiko von Abstoßungsreaktionen und erhöhter Mortalität verbunden zu sein [28, 29]. Vor allem Depressivität, Angst, negative Einstellungen und Erwartungen sowie längere Zeitspanne seit der Transplantation wurden in der bislang publizierten Literatur mit der medikamentösen Malcompliance in Verbindung gesetzt [19, 30]. Gemäß der aktuellsten AWMF-S3-Leitlinie ist eine psychosoziale Intervention u. a. zur Optimierung der Adhärenz empfohlen [19].

Ein multidisziplinäres Team ist in der Behandlung der Nierentransplantierten von essentieller Bedeutung. Nur mit einer guten Vorbereitung und engmaschige Nachbetreuung ist eine weitere Besserung der HRQoL bei Nierentransplantierten möglich. Im Vordergrund steht aktuell die Adhärenz zu den neusten publizierten Daten sowie Transplantationsrichtlinien und insbesondere die weitere Forschung und Entwicklung von qualitativ hochwertigen Interventionsstudien in diesem Bereich der Transplantationsmedizin. Die Nierentransplantation ist ökonomisch gesehen eine sehr aufwändige Therapie, wobei zusätzlich zu den finanziellen Aspekten rund um die Transplantation selber die regelmäßige Nachkontrollen einzuberechnen sind. Nicht nur aus medizinischen sowie auch aus wirtschaftlichen Gründen sollte ein komplikationsarmer Verlauf im Vordergrund stehen, mit möglichst hoher Kosteneffektivität und frühzeitiger Rückkehr in das Berufsleben der Betroffenen.

Aus der aktuellen Datenlage und der schnellen Evidenzsynthese ergeben sich konsequenterweise folgende Ideen für Interventionsstudien, um die HRQoL weiter zu verbessern und so das Outcome und die Effektivität der Nierentransplantation zu steigern:

1. bessere Adhärenz/Compliance an die immunsuppressive Medikation,
2. frühzeitiges Screening von kognitiver Verschlechterung nach Nierentransplantation mit dem Ziel von Verbesserung des neuro-kognitiven Status und Erhöhung der gesellschaftlichen Teilnahme,
3. frühzeitige Symptomkontrolle und aktives Einbringen des Patienten in die Lösungssuche, Entwicklung von Coping-Strategien bei den Patienten,
4. berufliche Rehabilitation und soziale Unterstützung, Wiedereingliederungsprogramme,
5. Optimierung von bestehenden Atemwegserkrankungen, ggf. in interdisziplinärer Zusammenarbeit mit der Pulmologie,
6. Untersuchungen zum Einfluss des Ernährungsverhaltens auf die HRQoL von Nierentransplantierten und ggf. Interventionen zur Gewichtsreduktion und Halten einer gesunden Mischdiät.

Zusätzlich müssen in diesen Studien weitere Einflussfaktoren identifiziert werden (z. B. Komorbiditäten des einzelnen Patienten).

Selbstverständlich ist auch diese Arbeit nicht ohne Limitationen, so muss diskutiert werden, dass die inkludierten Studien teils methodische Mängel aufweisen und ein Risiko für Bias besteht (bei 5 Studien moderates Risiko für Bias, bei einer Studie hohes Risiko für Bias). Außerdem handelt es sich lediglich um eine schnelle Evidenzsynthese.

Zu den Stärken dieser Arbeit gehört, dass die aktuelle Datenlage dargestellt wird. Es konnte klar gezeigt werden, dass es sich um ein Thema mit Datenlücken handelt, bei dem ein essentieller Bedarf an Interventionsstudien besteht. Eine weitere Stärke dieser Arbeit ist die klare Fokussierung auf die adulte Nierentransplantation mit Ausschluss von z. B. Kombinationstransplantationen.

Zusammenfassend ist es essentiell, die Lebensqualität der Nierentransplantierten weiter zu verbessern und so die Lebens- und Funktionsfähigkeit des Transplantats zu optimieren, was auch großen Einfluss auf die Kosteneffektivität im Gesundheitswesen haben könnte.

## Fazit für die Praxis

- Nierentransplantierte haben im Gegensatz zu terminal niereninsuffizienten Patienten ein besseres klinisches Monitoring der Lebensqualität (HRQoL).
- Es besteht das Potenzial, diese HRQoL weiter zu verbessern und damit das Outcome der Nierentransplantierten.
- Interventionsstudien zur Identifikation von Interventionen zur weiteren Verbesserung der HRQoL fehlen.
- Interventionsstudien sollten physische Symptome (z. B. Atemwegssymptome, Verbesserung der kognitiven Funktion, berufliche Rehabilitation und ernährungsmedizinische Aspekte) adressieren.
